# Translational Implications of The Gut Microbiome in Women with A Benign or Malignant Pelvic Mass

**Published:** 2025-02-12

**Authors:** Priya Sabu, Harsh B Pathak, Emily Nissen, Prabhakar Chalise, Devin C Koestler, Andrew K Godwin, Shahid Umar, Lori Spoozak, Andrea Jewell, Diane E Mahoney

**Affiliations:** 1 Division of Gynecologic Oncology, Department of Obstetrics and Gynecology, University of Kansas Medical Center, USA.; 2 Department of Pathology and Laboratory Medicine, University of Kansas Medical Center, USA.; 3 Kansas Institute for Precision Medicine, University of Kansas Medical Center, USA.; 4 Department of Biostatistics & Data Science, University of Kansas Medical Center, USA.; 5 University of Kansas Cancer Center, Kansas City, KS, USA.; 6 Department of Surgery, University of Kansas Medical Center, USA; 7 School of Nursing, University of Kansas Medical Center, USA

**Keywords:** Microbiome, Pelvic mass, Ovarian cancer, Microbiome diversity, Microbial composition, Clinical translation

## Abstract

**Objective:**

The role of the gut microbiome in non-gastrointestinal cancers has generated growing interest in the field of gynecologic oncology. Our objective was to characterize the gut microbiome in women with a pelvic mass suspicious for ovarian cancer. We hypothesized that (1) women with a pelvic mass would have reduced gut microbiota bacterial diversity compared to healthy controls and (2) gut microbial diversity would differ between benign disease compared to ovarian cancer.

**Methods:**

In this case-control observational study, patients who presented with a suspicious pelvic mass were recruited from university affiliated gynecologic oncology clinics for fecal biospecimen donation. Fecal samples that were obtained from patients underwent 16S rRNA gene sequencing for microbial evaluation and statistical analysis. We used the Human Microbiome Project (HMP) Data Portal to compare gut microbiota profiles for our study to that of healthy female controls.

**Results:**

Fifteen patients with a pelvic mass were included ages 24–75 years. When comparing the gut microbiomes of these patients to 82 healthy females from the HMP Dataset, those with a pelvic mass had a significantly lower microbiota gut bacterial diversity. On the final pathology, 8 of the 15 patients with a suspicious pelvic mass had ovarian cancer and 7 had benign disease. Although not statistically significant, the alpha diversity was marginally reduced in patients with ovarian cancer compared to those with benign disease.

**Conclusion:**

These findings underscore the necessity for validation in larger patient cohorts for clinical translation as a potential tool for disease diagnostics and disease prediction in diverse populations.

## Introduction

In the United States, ovarian cancer is one of the leading causes of death among women with gynecologic cancers [1]. The rate of new cases was 10 out of 100,000 women per year based on 2017–2021 cases and the mortality rate was 6 out of 100,00 women per year based on 2018–2022 deaths [2]. Unfortunately, the mortality rate of ovarian cancer remains steadily high as nearly 14,000 women succumb to the disease annually [3]. As there is an urgency to better understand the etiology of ovarian cancer, growing attention is being directed to the relationship between the human microbiome and the development of ovarian cancer [4–6].

The most common type of ovarian cancer is of epithelial histology, accounting for close to 90% of cases [7]. Studies have linked gut microbiota dysbiosis to the development of Epithelial Ovarian Cancer (EOC) [8,9]. Since the Human Microbiome Project (HMP) launched in 2007, significant research efforts have been ongoing to examine the role of the human microbiome in disease and to comprehend possible clinical implications [10]. The human microbiome is the collective genomes of microbes (bacteria, viruses, fungi) in humans that may hold a key role in ovarian cancer detection, development, disease severity, progression, clinical symptoms, and response to treatment [11–13]. Humans have a symbiotic relationship with the microbiome that can be influenced by social, behavioral, environmental, hormonal, and genetic factors. Therefore, alterations in the human microbiome homeostasis could exacerbate disease states [14–16].

While definitive mechanisms of the human microbiome in gynecological cancers remain under investigation, researchers have described microbiota bacterial differences in site-specific tissue including the gut of patients with EOC compared to those with benign disease [17–20]. An animal model study illustrated that altering the microbiome with various antibiotic cocktails altered tumorigenesis in mice with high-grade serous ovarian cancer, an aggressive EOC subtype [21]. Additionally, the peritoneal microbial composition has been distinctly unique between ovarian cancer patients and those with a benign pelvic mass [22]. Corroborating evidence suggests that variations in microbial composition could be an influential factor in the disease initiation, progression, and treatment response [23,24].

The objective of the study was to characterize the gut microbiome in women with a pelvic mass suspicious for ovarian cancer. We hypothesized that patients presenting with a pelvic mass would have reduced gut microbiota bacterial diversity compared to healthy controls. Additionally, we postulated gut microbiota bacterial diversity would be further differentiated between women with ovarian cancer compared to those with benign disease confirmed on tissue histology.

## Methods

### Study population

This was a case-control observational study in which patients were recruited from gynecologic oncology clinical offices located in the Midwestern region of the United States. Human subjects approval was obtained by the University of Kansas Medical Center Institutional Review Board (IRB) under the existing Biospecimen Repository Core Facility (BRCF) protocol (HSC #5929). Study coordinators approached women who met the study criteria during the office visit or surgical appointment for fecal sample donation. The study coordinators obtained written informed consent from each patient participant prior to study enrollment. Participants were provided a specialized fecal self-collection home kit (Omnigene Gut; DNA Genotek, Ontario, Canada) with instructions on use and were asked to return fecal samples to the BRCF for storage at −80°C.

### Study inclusion criteria

Inclusion criteria were: (1) suspected or confirmed initial diagnosis of ovarian cancer, (2) aged 18 years and older, (3) patients being able to collect fecal samples, and (4) ability to read and write in English. Patients with gastrointestinal malignancies, inflammatory bowel diseases, or current use (within the past four weeks) of antibiotics were excluded. A full description of the inclusion and exclusion criteria is provided in Table 1.

### Human samples

All participant returned fecal samples were frozen and stored at the BRCF until the microbial DNA extraction process. Approximately 200 milligrams of the fecal sample were used for microbial DNA extraction using the QIAamp FAST DNA Fecal Mini kit (QIAGEN, Hilden, Germany) per manufacturer’s guidelines. We assessed the yield of the extracted genomic DNA (gDNA) from the fecal samples using the PicoGreen^®^ fluorescence assay. The quality of gDNA was evaluated using DNA TapeStation (Agilent) assessment. The microbial 16s rRNA gene sequencing was performed by The University of Kansas Genome Sequencing Core (Lawrence, KS) on the Illumina MiSeq platform in the V3-V4 region.

### Bioinformatic processing

The 16S rRNA sequence read quality was assessed with FastQC (v0.11.8; http://www.bioinformatics.babraham.ac.uk/projects/fastqc/). A quality trim was performed using Trimmomatic (v.0.39; http://www.usadellab.org/cms/?page=trimmomatic) using a sliding window approach (window size 4–15) to remove bases off the end of a read if the average quality within the window falls below a quality of 20. Data for the healthy controls were obtained from the HMP Data Portal (https://portal.hmpdacc.org/). Specifically, we used the specific parameters, Projects = Human Microbiome Project, Body Site = feces, Studies = Healthy Human Subjects (HHS), and Gender = female. We downloaded the trimmed sequence data (Format = FASTA and Type = 16s_trimmed_seq_set in the portal) for samples that matched the above criteria (N=82). The 16S rRNA sequencing for the HMP was performed using the Roche-454 FLX Titanium platform and the V3-V5 variable region window was sequenced for all samples (see https://hmpdacc.org/hmp/micro_analysis/microbiome_analyses.php for more details on HMP metagenomic and analysis sequencing details).

QIIME2 was used for further processing and analysis of the data. Paired FASTQ files for the women with a pelvic mass (n=15) were first imported into QIIME2 [25]. The first 13 bases of the forward reads and the first 5 bases of the reverse reads were trimmed. Forward reads were also truncated at 280 bases and reverse reads were truncated at 240 bases. Samples were also denoised and dereplicated using the dada2 denoise-paired tool. The prior trimmed FASTA files from the HMP were then imported into QIIME2 and a merged feature table was created to combine the two datasets. Closed-reference clustering with the merged data was performed by clustering reads against the Greengenes 13_8 99% Operational Taxonomic Units (OTUs) full-length sequences reference sequence collection. Any reads which did not hit a sequence in the reference collection were excluded from downstream analysis. The phylogenetic tree was built using the Fasttree program [26,27].

### Data analysis

#### Microbial diversity indices:

Alpha diversity (α-diversity) is defined as the mean diversity of species in different sites or habitats within a local scale. We used the following indices to assess the α-diversity in our samples: (1) Shannon’s entropy (also known as Shannon’s diversity index), (2) Phylogenetic diversity (Faith PD, which uses phylogenetic distance to calculate the diversity of a given sample), and (3) Evenness which refers to how close in numbers each species is in an environment [28]. The different measures reflect the richness (number) or distribution (evenness) of a microbial sample or aim to reflect a combination of both properties.

Differences in each of the α-diversity metrics between healthy women and women with a pelvic mass were assessed using the Wilcoxon ranked sum test. Tests were considered statistically significant if the p-value was <0.05. Beta-diversity (β-diversity) is defined as the ratio or distance between two or more different sites or habitats. We used the following β-diversity indices: Jaccard distance, Bray-Curtis distance, Unweighted UniFrac distance, and Weighted UniFrac distance [29]. Principal Coordinates Analysis (PCoA) was used to visualize sample similarities. Permutational multivariate analysis of variance (PERMANOVA) models were used for each of the β-diversity metrics to compare healthy women and women with a pelvic mass. Differential abundances of the microbiome between the groups were assessed using ANCOM (analysis of the composition of microbiomes) models [30]. ANCOM was used to detect differences in microbial mean taxa abundance between healthy controls (n=82) and women with a pelvic mass (n=15). ANCOM accounts for the underlying compositional structure of microbiome data (e.g., compositional, in nature) and has been shown to outperform competing methods in terms of controlling False Discovery Rate (FDR) under the nominal level (commonly 5%) while maintaining the statistical power. Analyses were conducted at the following levels: Level 2 (Phylum level), Level 6 (Genus level), and Level 7 (Species level) [31].

## Results

A total of 15 patients were enrolled into the study who presented with a suspicious pelvic mass. Ovarian cancer was documented on final pathology report in 8 of the 15 patients. Malignant histology included high-grade serous carcinoma, granulosa cell, endometrioid carcinoma, carcinosarcoma, adenocarcinoma, and sarcomatoid carcinoma. Types of benign ovarian histology included serous cystadenoma, Brenner tumor, mature cystic teratoma, endometriosis cyst, mucinous borderline, benign ovarian tissue, borderline seromucinous, leiomyoma, and serous cystadenofibroma. Fourteen out of 15 patients had a family history of malignancy and 11 out of 15 patients were post-menopausal women. Patient clinical and demographic data extracted from the electronic medical record are summarized in Table 2 by benign disease versus ovarian malignancy.

**Figure 1:**
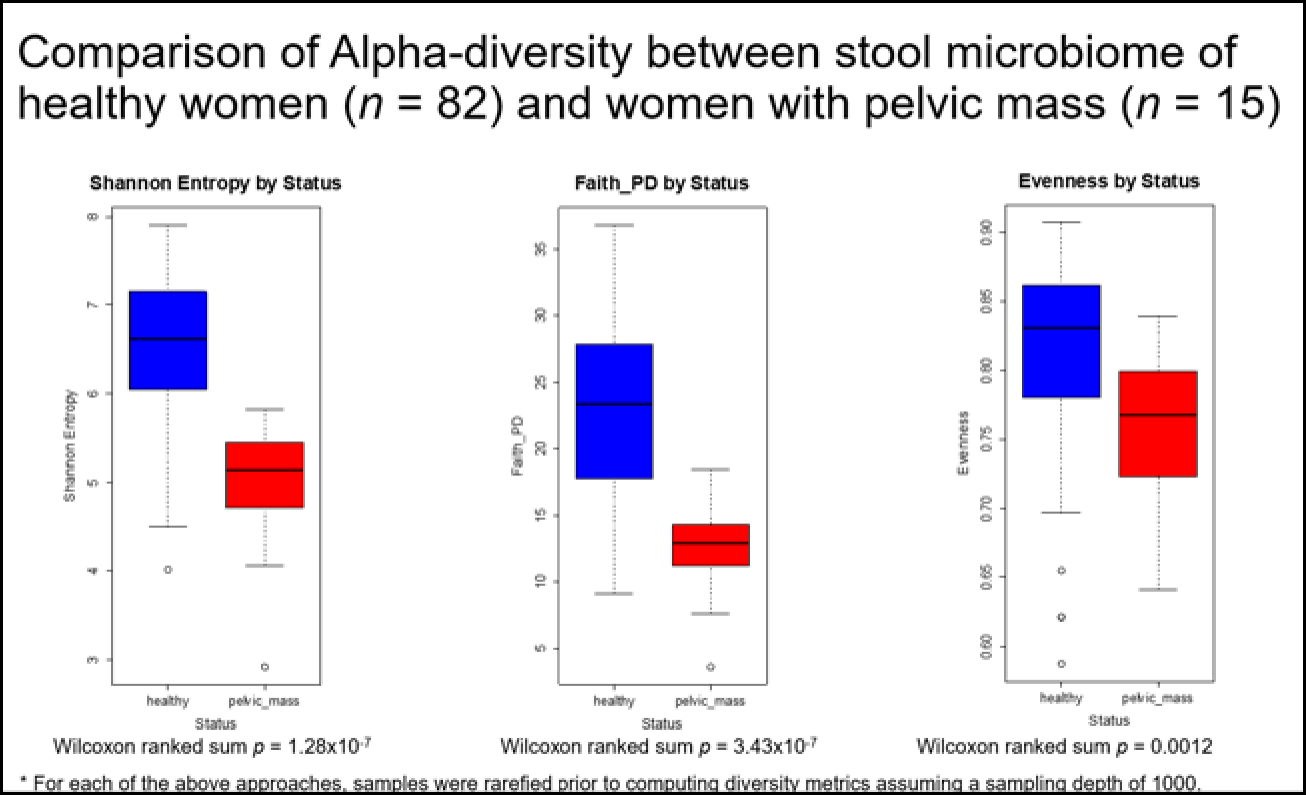
Alpha-diversity of fecal microbiome in healthy controls (n=82) vs. women with pelvic mass (n=15). Shown are comparisons by Shannon’s index, Phylogenetic Diversity (PD) and Evenness (commonly used measures of bacterial richness within samples). A p-value of <0.05 indicates statistical significance.

We compared the α-diversity between the fecal microbiome of healthy women from the Human Microbiome Project (HMP) (n=82) and women with a pelvic mass (n=15) who were prospectively recruited. We observed that those with a pelvic mass had a statistically significant lower microbiome diversity than the healthy control across all alpha diversity indices (Shannon entropy p-value = 0.0000001, Faith phylogenetic diversity p-value = 0.0000003, Evenness p-value = 0.0012) (Figure 1).

**Figure 2:**
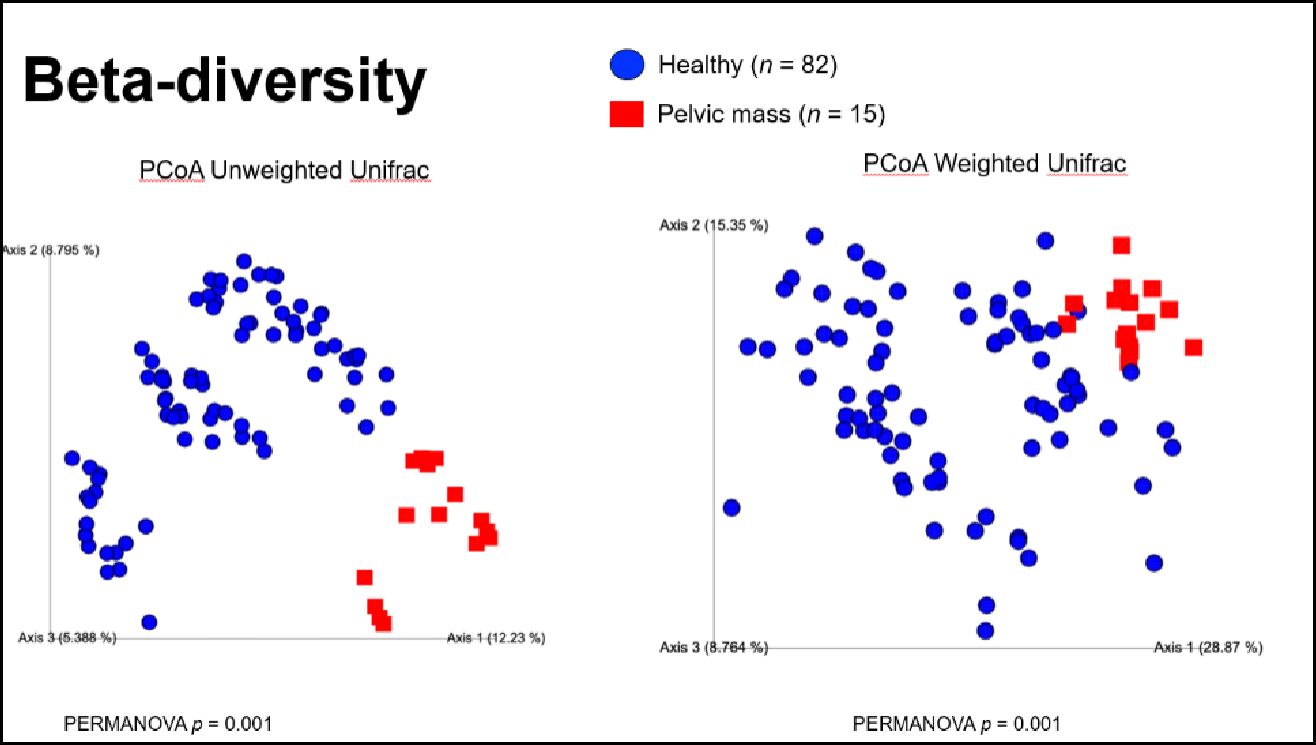
Beta-diversity of fecal microbiome in healthy controls (n=82) vs. women with pelvic mass (n=15) Shown are comparisons by Unweighted and Weighted Unifrac distance (commonly used measures of bacterial uniqueness between samples). A p-value of <0.05 indicates statistical significance.

**Figure 3:**
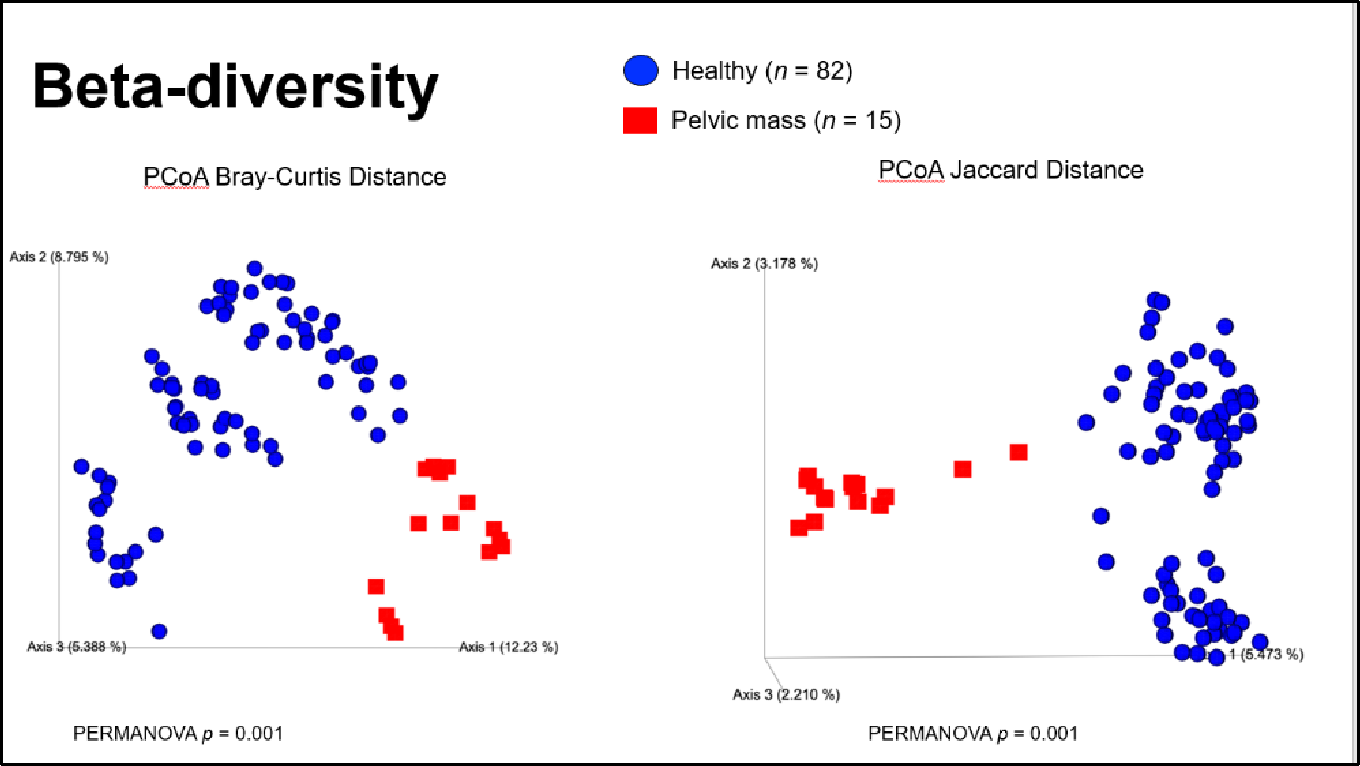
Beta-diversity of fecal microbiome in healthy controls (n=82) vs. women with pelvic mass (n=15). Shown are comparisons by Bray-Curtis and Jaccard (commonly used measures of bacterial uniqueness between samples). A p-value of <0.05 indicates statistical significance.

Beta diversity metrics were used to compare how distinct the fecal microbiome was between healthy women and women with a pelvic mass. Across all metrics, we observed that the fecal microbiome was compositionally distinct between the two groups (Jaccard p-value = 0.001, Bray-Curtis p-value = 0.001, Unweighted UniFrac p-value = 0.001, Weighted Unifrac p-value = 0.001) (Figures 2 & 3).

Differential abundance analyses were conducted on the phylum, genus, and species levels. There were 12 OTUs that were found to be significantly differentially abundant between fecal samples from healthy women and those with a pelvic mass. Eight OTUs were more abundant in healthy controls and 4 OTUs were more abundant in women with a pelvic mass (Table 3).

Table 3 Differential Abundance of bacteria in Healthy Controls compared to Women with a pelvic mass. Shown are 12 Operational Taxonomic Units (OTUs) differentially abundant in fecal samples of women with a suspicious pelvic mass compares to healthy controls. (OTUs categorize bacterial clusters by DNA sequence similarity).

**Figure 4:**
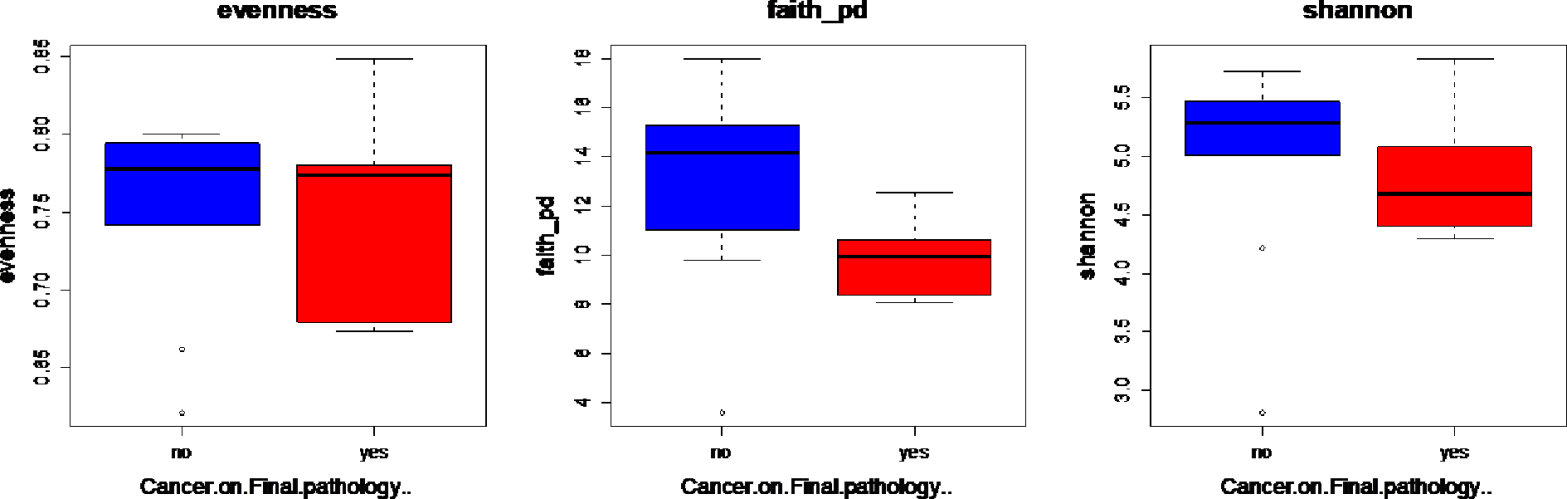
Alpha diversity in women with ovarian cancer vs. benign disease. For each of the above approaches, samples were rarefied prior to computing diversity metrics assuming a sample depth of 1000. Evenness – Wilcoxon ranked sum p=0.9530; Faith-Wilcoxon ranked sum p=0.0553; and Shannon-Wilcoxon ranked sum p=0.5135.

Lastly, we compared the fecal microbiome of women with a pelvic mass with respect to the following variables: (1) Cancer on final pathology, (2) smoking status, and (3) BMI. The alpha diversity using the Faith_PD was marginally lower among women with cancer on final pathology although not statistically significant. (Figure 4) Moreover, the overall α-diversity did not appear to be significantly associated with smoking status or BMI. None of the β-diversity metrics were significantly associated with cancer on final pathology. Thus, there were no differentially abundant bacteria identified based on cancer on final pathology.

## Discussion

The objective of this study was to identify differences in gut microbiota bacterial diversity that may be associated with a pelvic mass suggestive of ovarian malignancy. Our data demonstrates a relationship between the gut microbiome and the presence of a pelvic mass potentially warranting translation for clinical application. Often times, women who are diagnosed with ovarian cancer initially present in gynecologic oncology settings requiring a clinical workup with for a pelvic mass based on imaging results suspicious for malignancy. Therefore, the utilization of gut microbiota-based tests could serve as complementary to existing tools if found to be reliable. The International Ovarian Tumor Analysis (IOTA) group has incorporated ultrasound findings to help predict the risk of malignancy of a pelvic mass before surgery. These rules can be applied to about 75% of cases with a 90% sensitivity and 95% specificity [32–34]. More recently, the IOTA group expanded the model to predict type and stage of ovarian malignancy and determine the treatment plan [35]. We integrated the IOTA model into our inclusion criteria to assist us in selecting patients who would most likely have concerns for malignancy. Our study findings raise the question if certain gut bacterial strains would complement the IOTA risk assessment for differentiating pelvic masses. Researchers reported that fecal microbes *Corynebacterium, Dialister*, *Peptoniphilus*, and *Prevotella* have differentiated early versus late-stage disease [5].

We found that women with a suspicious pelvic mass were noted to have a distinct abundances of gut bacteria and a reduced bacterial diversity when compared to healthy women. Irrespective of the methods used to evaluate the alpha diversity, we noted that the fecal samples from healthy women were significantly more diverse than fecal samples from women with a pelvic mass suspicious for malignancy. When examining the beta diversity, the results showed that the fecal samples from healthy women are compositionally distinct from women with a pelvic mass. These data suggest that changes in the gut microbiome may predispose patients to the development of pelvic pathology or be a consequence of such changes. Consistent with our findings, prior studies have shown differences in gut microbial profiles of women with malignant and non-malignant pathology that were distinctly different compared to controls among ovarian and breast cancer populations [36,37]. Furthermore, gut microbial dysbiosis has resulted in accelerated tumor growth in mice and platinum chemotherapy resistance in ovarian cancer patients and animal models [8,24,38].

The normal gut microbiome is mainly comprised of Gram negative *Bacteriodetes* and Gram positive *Firmicutes* and *Actinobacteria.* A high abundance of *Proteobacteria* was observed in gut dysbiosis and was identified as an indicator of intestinal epithelial dysfunction [12]. Studies examining ovarian cancer oncobiome and its’ signature patterns have noted an increased ratio of *Proteobacteria/Firmicutes* in ovarian cancer tissue as compared to healthy tissue [17,18]. We hypothesized that patients with ovarian cancer on the final pathology would have decreased fecal microbial diversity and a shift in the microbiome towards pathogenic strains in comparison to patients with benign pathology. While our study demonstrated marginal differences in the fecal α-diversity among women with ovarian malignancy compared to those with benign disease, we did not detect a shift of the microbiome towards pathogenic strains. We believe that a larger sample size may have resulted in more pronounced findings. Although there has been limited research assessing the fecal microbiome in patients with a pelvic mass, one study observed significant differences in the β-diversity of the bacteria in the fecal samples when comparing women with ovarian cancer (n=40) to those with benign disease (n=40). Specifically, the authors report that *Akkermansia* genus was reduced in women with ovarian malignancy [39].

One of the limitations of our study is generalizability of results based on our small sample size. Our analysis included 8 patients with cancer on final pathology and 7 patients with benign pathology. Additionally, the histologic characteristics were heterogenous in both the groups of women with cancer on final pathology and with benign pathology that limited our ability to detect a pattern of statistical significance. Detecting a marginal difference in the groups suggests that clearly identifying a relationship in a larger sample size with specific histologic types may help us further characterize differences. There was limited information on the healthy control women from the HMP. The samples were collected more than a decade ago, and there was no information available on their age or clinical characteristics. Additionally, the sequencing platform used by the HMP was Roche-454 FLX Titanium and the platform used in this study was Illumina MiSeq. The sequenced regions evaluated by the HMP and this study may have involved overlapping hypervariable regions.

## Conclusion

In conclusion, the gut microbiome in women with a pelvic mass was found to be compositionally distinct from the healthy women. These findings suggest that changes in the gut microbiome may predispose patients to the development of pelvic pathology or be a consequence of those changes. Women with ovarian malignancy had a marginally reduced diversity of fecal bacteria when compared to women with benign pelvic masses warranting additional investigation. While there are no current recommended universal screening tests for ovarian cancer, the human microbiome could hold profound implications in predicting women who are most at risk for ovarian malignancy and benign disease.

## Figures and Tables

**Table 1.** Study Protocol Inclusion and Exclusion criteria

Inclusion criteria	Exclusion criteria
**1.** Greater than or equal to 18 years of age **2.** Suspected epithelial ovarian cancer **a.** Elevated CA-125 (tumor marker) **b.** Abnormal imaging **i.** Pelvic mass includes the presence of both solid and cystic areas within a lesion; necrosis within a solid lesion; papillary projections from the wall or septum of a cystic lesion; an irregular septum or wall; multiple thickened (>3 mm) septations; a large size (>6 cm); bilateral lesions; and ascites, peritoneal disease, or lymphadenopathy **ii.** Other possible words in imaging results: large pelvic or ovarian mass, omental caking, or peritoneal carcinomatosis **3.** Confirmed ovarian cancer diagnosis via tissue or cytology evaluation **4.** Any tumor stage according to the International Federation of Gynecology and Obstetrics (FIGO) **5.** Chemotherapy naive	1. Gastrointestinal malignancies 2. irritable bowel syndrome 3. inflammatory bowel disease 4. currently taking antibiotics 5. pregnant and/or lactating

**Table 2.** Patient Demographics

	Benign (n= 7)	Ovarian Cancer (n=8)
Age range	28–75 y/o	24–75 y/o
Body Mass Index (BMI), range	20–30	18–40
History of Diabetes, N (%)	2 (28.6)	3 (37.5)
History of Sexually Transmitted Diseases, N (%)	0 (0)	0 (0)
Engages in routine physical activity, N (%)	0 (0)	1 (12.5)
Current cigarette smoker, N (%)	6 (85.7)	2 (25.0)
Current alcohol use, N (%)	5 (71.4)	2 (25.0)
Current illicit drug use, N (%)	1 (14.3)	0 (0)
Post-menopausal, N (%)	5 (71.4)	7 (87.5)
Nulliparous, N (%)	3 (42.9)	0 (0)
Family history of malignancy, N (%)	7 (100)	7 (87.5)
Prior history of cancer (any type), N (%)	0 (0)	2 (25.0)

**Table 3.** Differential Abundance of Operational Taxonomic Units (OTUs) in Healthy Controls compared to women with a pelvic mass

#	Name of OTU	Median # of mapped OTUs in Healthy patients	Median # of mapped OTUs in women with a pelvic mass	Relative abundance in women with a pelvic mass
1	**Bacteroidetes**	41	1	Decreased
2	**Bacteroidetes; Bacteroidia**	13	1	Decreased
3	**Bacteroidetes; Bacteroidia; Bacteroidales**	44	1	Decreased
4	**Bacteroidetes; Bacteroidia; Bacteroidales; Rikenellaceae**	21.5	1	Decreased
5	**Firmicutes; Clostridia; Clostridiales; Ruminococcaceae;Faecalibacterium**	14	1	Decreased
6	**Firmicutes; Clostridia; Clostridiales; Lachnospiraceae; Clostridium; lavalense**	1	81	Increased
7	**Bacteroidetes; Bacteroidia; Bacteroidales; Porphyromonadaceae**	14	1	Decreased
8	**Firmicutes; Clostridia; Clostridiales; Ruminococcaceae; Subdoligranulum; variabile**	12.5	1	Decreased
9	**Firmicutes; Clostridia; Clostridiales; Lachnospiraceae; Clostridium; citroniae**	1	90	Increased
10	**Firmicutes; Clostridia; Clostridiales; Ruminococcaceae; Anaerotruncus**	1	34	Increased
11	**Proteobacteria; Deltaproteobacteria; Desulfovibrionales; Desulfovibronaceae; Bilophila**	1	107	Increased
12	**Firmicutes**	5	1	Decreased
